# SNHG22 overexpression indicates poor prognosis and induces chemotherapy resistance via the miR-2467/Gal-1 signaling pathway in epithelial ovarian carcinoma

**DOI:** 10.18632/aging.102313

**Published:** 2019-10-03

**Authors:** Peng-Fei Zhang, Jing Wu, Jin-Hong Luo, Ke-Sang Li, Fei Wang, Wei Huang, Yin Wu, Shui-Ping Gao, Xue-Mei Zhang, Peng-Nan Zhang

**Affiliations:** 1 Department of Oncology, Shanghai East Hospital, Tongji University School of Medicine, Shanghai, China; 2 Department of Hematology and Oncology, Hwa Mei Hospital, University of Chinese Academy of Sciences, Ningbo, China; 3 Department of Gynecology, Obstetrics and Gynecology Hospital of Fudan University, Shanghai Key Laboratory of Female Reproductive Endocrine Related Diseases, Shanghai, China

**Keywords:** long-noncoding RNA, epithelial ovarian carcinoma, galectin-1, chemoresistance

## Abstract

Recently, an increasing number of studies have reported that dysregulation of long noncoding RNAs (lncRNAs) plays an important role in cancer initiation and progression, including in epithelial ovarian carcinoma (EOC). However, little is known about the detailed biological functions of the lncRNA small nucleolar RNA host gene 22 (SNHG22) during the progression of EOC. Here, we found that SNHG22 was significantly increased in EOC tissues and was significantly associated with a low level of differentiation. Forced SNHG22 expression promoted chemotherapy resistance in EOC cells. Knockdown of SNHG22 expression increased the sensitivity of EOC cells to cisplatin and paclitaxel. Importantly, we found that SNHG22 could directly interact with miR-2467 and lead to the release of miR-2467-targeted Gal-1 mRNA. Moreover, SNHG22 overexpression induced EOC cell resistance to chemotherapy agents via PI3K/AKT and ERK cascade activation. In summary, our findings demonstrate that SNHG22 plays a critical role in the chemotherapy resistance of EOC by mediating the miR-2467/Gal-1 regulatory axis.

## INTRODUCTION

Epithelial ovarian carcinoma (EOC) is the most common gynecological cancer and the fifth leading cause of cancer-related deaths in women [1]. The poor prognosis results from its high recurrence following curative resection, distant metastasis, and resistance to systemic chemotherapy [2–4]. Cisplatin and paclitaxel-based chemotherapies are first-line treatment regimens for most advanced and relapsed EOC patients; however, primary and secondary resistance to these therapeutics have been a major obstacle in EOC therapy [3, 5, 6]. Therefore, more detailed studies are required to explore the molecular mechanisms of drug resistance associated with EOC chemotherapy.

Long noncoding RNAs (lncRNAs) are a class of RNAs longer than 200 nucleotides without protein-coding potential. Dysregulation of lncRNA expression has been found in almost all human tumors, providing numerous promising diagnostic biomarkers and therapeutic targets [7]. Recently, several lncRNAs, including small nucleolar RNA host genes (SNHGs), including SHNG1, SNHG3, SNHG4, SNHG5, SNHG6, SNHG7, SNHG8, SNHG10, SNHG12, SNHG14, and SNHG15, have been reported to act as oncogenes, participating in tumorigenesis and progression [8–18]. For example, increased SNHG3 expression is correlated with poor prognosis and sorafenib resistance in hepatocellular carcinoma (HCC) [9]. Forced SNHG7 expression upregulated N-acetylgalactosaminyltransferase 1 (GALNT1) expression through sponging miR-216b, thus playing an oncogenic role in colorectal cancer (CRC) [19]. Moreover, SNHG7 also played the oncogenic role in regulating PI3K/AKT/mTOR pathway by acting as a competing endogenous RNA (ceRNA) for acetylgalactosaminyltransferase 7 (GALNT7) in CRC [13]. However, the biological role of SNHGs in cancer cells remains poorly understood. For example, the expression profile and function of some SNHGs in cancers have not been reported, including those of SNHG2 and SNHG22. SNHG22 is a recognized lncRNA and is located on chromosome 18q21.1. Our preliminary results found that the levels of SNHG22 were upregulated in EOC tissues; therefore, we explored the expression and function of SNHG22 in EOC cells. Galectin-1 (Gal-1) is considered one of the representative galectins and is upregulated in many cancers, including EOC [5]. Our previous studies have confirmed that forced Gal-1 expression induces cancer resistance to anti-tumor agents and is associated with poor prognosis in HCC and EOC [5, 20, 21].

Here, we found that high SNHG22 expression was associated with poor prognosis in EOC patients. SNHG22 silencing increased the sensitivity of EOC cells to cisplatin and paclitaxel, while SNHG22 overexpression promoted EOC cell resistance to cisplatin and paclitaxel. Furthermore, we revealed that SNHG22 promoted EOC chemotherapy resistance by sponging miR-2467 and acting as a ceRNA for Gal-1. Overall, our findings suggest that SNHG22 could be a promising therapy target in EOC.

## RESULTS

### SNHG22 is overexpressed in EOC tissues and correlates with poor prognosis in EOC patients

**Figure 1:**
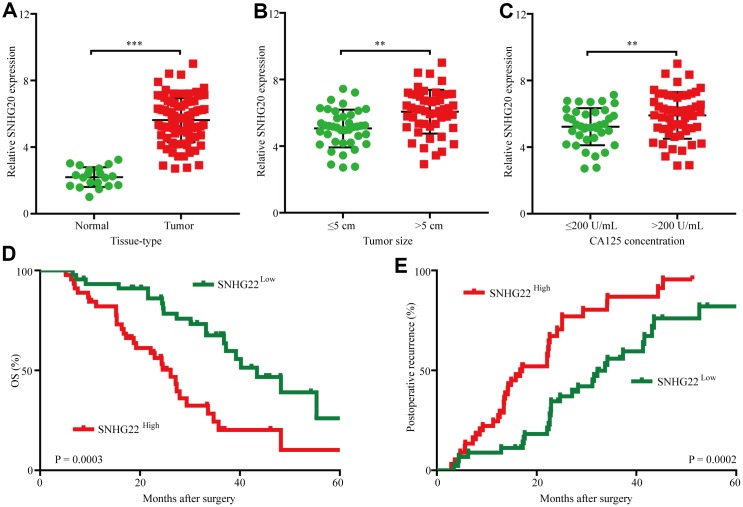
**The upregulation of SNHG22 predicts poor outcome in EOC patients.** (**A**) Expression levels of SNHG22 in 90 cases of EOC patient tissues and 20 cases of normal ovarian tissues. (**B**) Ninety patients were divided into ≤5 cm and >5 cm size groups. The diagram shows SNHG22 expression in each group. (**C**) Ninety patients were divided into ≤200 U/mL and >200 U/mL CA125 groups. The diagram shows SNHG22 expression in each group. (**D** and **E**) Kaplan-Meier analysis of overall survival and recurrence in 90 patients with EOC according to SNHG22 expression (log-rank test). Data are presented as the means ± SDs in three independent experiments. **P < 0.01, ***P < 0.001.

To assess SNHG22 expression in EOC, we first analyzed SNHG22 expression in 90 cases of EOC tissues and 20 cases of normal ovarian tissues by qRT-PCR. The results revealed that SNHG22 expression in EOC tissues was higher than that in normal ovarian tissues (Figure 1A). Next, we investigated the relationship between SNHG3 expression and clinicopathological characteristics in 90 EOC patients, as listed in Table 1. The results demonstrated that compared with EOC patients with low SNHG22 expression, EOC patients with high SNHG22 expression had larger tumor sizes (P=0.001) and elevated CA125 expression (P=0.020) (Figure 1B and 1C). Then, we examined the prognostic implication of SNHG22 expression in EOC patients. Importantly, the results showed that patients with SNHG22^high^ expression had a significantly worse prognosis than those with SNHG22^low^ expression (Figure 1D and 1E). Multivariate analysis confirmed SHNG22 expression as an independent predictor for postoperative recurrence and overall survival (OS; Tables 2 and 3). These results indicate that SNHG22 likely participates in the progression of EOC.

**Table 1:** Correlation between SNHG22 and clinicopathological characteristics in 90 EOCs.

**Variable**	**SNHG22**		**P value**
	**Low**	**High**	
**Age (years)**			
≤50	21	19	0.832
>50	24	26	
**Stage**			
I-II	27	21	0.540
III-IV	23	24	
**CA125 in serum (U/mL)**			
<200	30	18	0.020
≥200	15	27	
**Differentiation**			
Low & Moderate	16	21	0.392
Positive	29	24	
**Lymph nodes metastasis**			
Negative	19	17	0.830
Positive	26	28	
**Tumor size (diameter, cm)**			
≤5	30	13	0.001
>5	15	32	

OS, overall survival; NA, not adopted; NS, not significant; 95%CI, 95% confidence interval; HR, hazard ratio; Cox proportional hazards regression model.

**Table 2:** Univariate and multivariate analyses of factors associated with overall survival.

**Factors**	**OS**			
	**Univariate, P**	**Multivariate**		
		**HR**	**95% CI**	**P value**
Age (years) (≤50 vs. >50)	0.278			NA
Stage (I-II vs. III-IV)	0.547			NA
CA125 in serum (U/mL) (<200 vs. ≥200)	0.032			NS
Differentiation (Low & Moderate vs. High)	0.625			NA
Lymph nodes metastasis (Negative vs. Positivele)	0.271			NA
Tumor size (diameter, cm) (>5 vs. ≤5)	0.014			NS
SNHG22 (high vs. low)	0.003	0.932	1.125–2.754	0.021

**Table 3:** Univariate and multivariate analyses of factors associated with cumulative recurrence.

**Factors**	**OS**			
	**Univariate, P**	**Multivariate**		
		**HR**	**95% CI**	**P value**
Age (years) (≤50 vs. >50)	0.397			NA
Stage (I-II vs. III-IV)	0.562			NA
CA125 in serum (U/mL) (<600 vs. ≥600)	0.744			NA
Differentiation (Low & Moderate vs. High)	0.612			NA
Lymph nodes metastasis (Negative vs. Positivele)	0.715			NA
Tumor size (diameter, cm) (>5 vs. ≤5)	0.006			NS
SNHG22 (high vs. low)	0.005	0.722	0.941–2.307	0.037

NA, not adopted; NS, not significant; 95%CI, 95% confidence interval; HR, hazard ratio; Cox proportional hazards regression model.

### SNHG22 promotes EOC cell chemotherapy resistance *in vitro*

**Figure 2:**
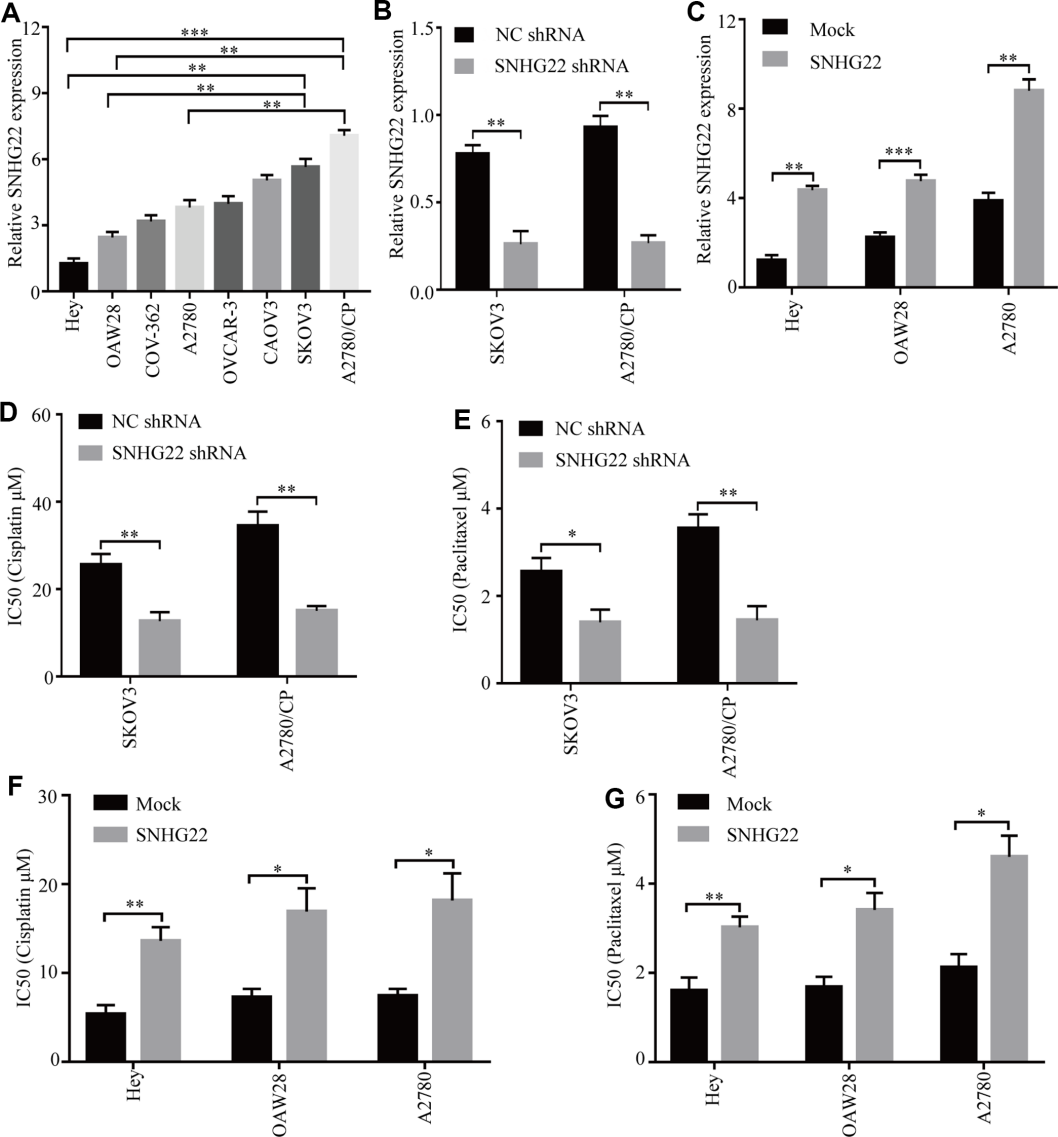
**High SNHG22 expression promotes EOC cell chemoresistance *in vitro*.** (**A**) SNHG22 expression in several EOC cell lines was examined using qRT-PCR analysis. GAPDH was used as an internal loading control. (**B**) SNHG22 expression in EOC SKOV3 and A2780/CP cells was modified by shRNA transfection. (**C**) SNHG22 expression in EOC Hey and OAW28 cells was modified by mock-lentivirus or SNHG22-lentivirus transfection. (**D**–**G**) Cisplatin or paclitaxel sensitivity was measured using CCK-8 assays in EOC cells. Data are presented as the means ± SDs in three independent experiments. *P < 0.05, **P < 0.01, ***P < 0.001.

The expression levels of SNHG22 in the human cell lines EOC OVCAR-3, OAW28, COV-362, SKOV3, CAOV3, Hey, and A2780 and the cisplatin-resistant EOC cell line A2780/CP were analyzed. The qRT-PCR results showed that the levels of SNHG22 in the A2780/CP cell line were significantly higher than those in the A2780 cell line (Figure 2A). These results indicate that SNHG22 is likely involved in the chemotherapy resistance of EOC cells. To further detect the biological function of SNHG22 in EOC cells, lentiviruses expressing SNHG22 or SNHG22 shRNA were constructed, and transfection efficiencies were confirmed using qRT-PCR (Figure 2B and 2C). The CCK-8 assay revealed that the A2780/CP-SNHG22 shRNA and SKOV3-SNHG22 shRNA groups had significantly lower IC50 values compared with the control cells treated with cisplatin or paclitaxel (Figure 2D and 2E). In contrast, the IC50 values in the Hey-SNHG22, OAW28-SNHG22, and A2780-SNHG22 groups were significantly increased compared with those seen in the control group treated with cisplatin or paclitaxel (Figure 2F and 2G).

### SNHG22 expression is inversely correlated with clinical EOC sensitivity to cisplatin and paclitaxel

**Figure 3:**
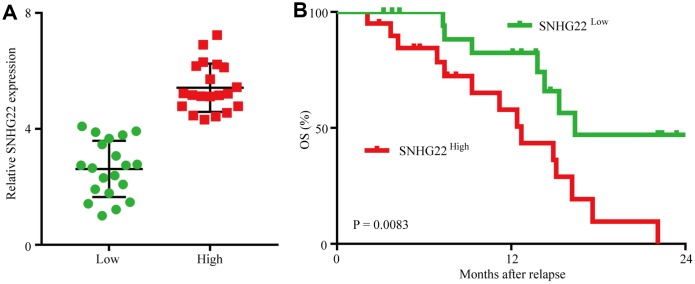
**Forced SNHG22 expression correlates with chemoresistance in EOC patients.** (**A**) A total of 40 patients were divided into SNHG22^low^ and SNHG22^high^ groups. The diagram shows the SNHG22 expression of each group. (**B**) Comparison of OS curves for patients with high and low SNHG22 expression that were treated with cisplatin or paclitaxel.

We then analyzed retrospective data from 40 advanced recurrent EOC patients receiving combined cisplatin and paclitaxel therapy who had undergone ovarian resection 2-60 months before combined chemotherapy. SNHG22 expression levels in EOC tissues were measured using qRT-PCR, and Kaplan-Meier survival analysis indicated that the OS probability for the SNHG22^high^ group was lower than that for the SNHG22^low^ group (Figure 3A and 3B). The median OS was 9.0 months in the SNHG22^high^ group and 14.0 months in the SNHG22^low^ group (SNHG22^high^ group hazard ratio 2.879; 95% confidence interval, 1.019–6.271; P<0.05); therefore, we speculate that forced SNHG22 expression leads to EOC resistance to cisplatin and paclitaxel.

### SNHG22 functions as a ceRNA to regulate Gal-1 expression in EOC

Recently, an increasing number of studies have reported that lncRNAs could serve as ceRNA sponges of miRNAs in several malignant tumors [10, 22, 23]. To explore potential target miRNAs of SNHG22, Starbase3.0 was used, and the results revealed 27 predicted target miRNAs. Our previous study reported that increased Gal-1 expression promotes chemoresistance to cisplatin in EOC cells [5].

**Figure 4:**
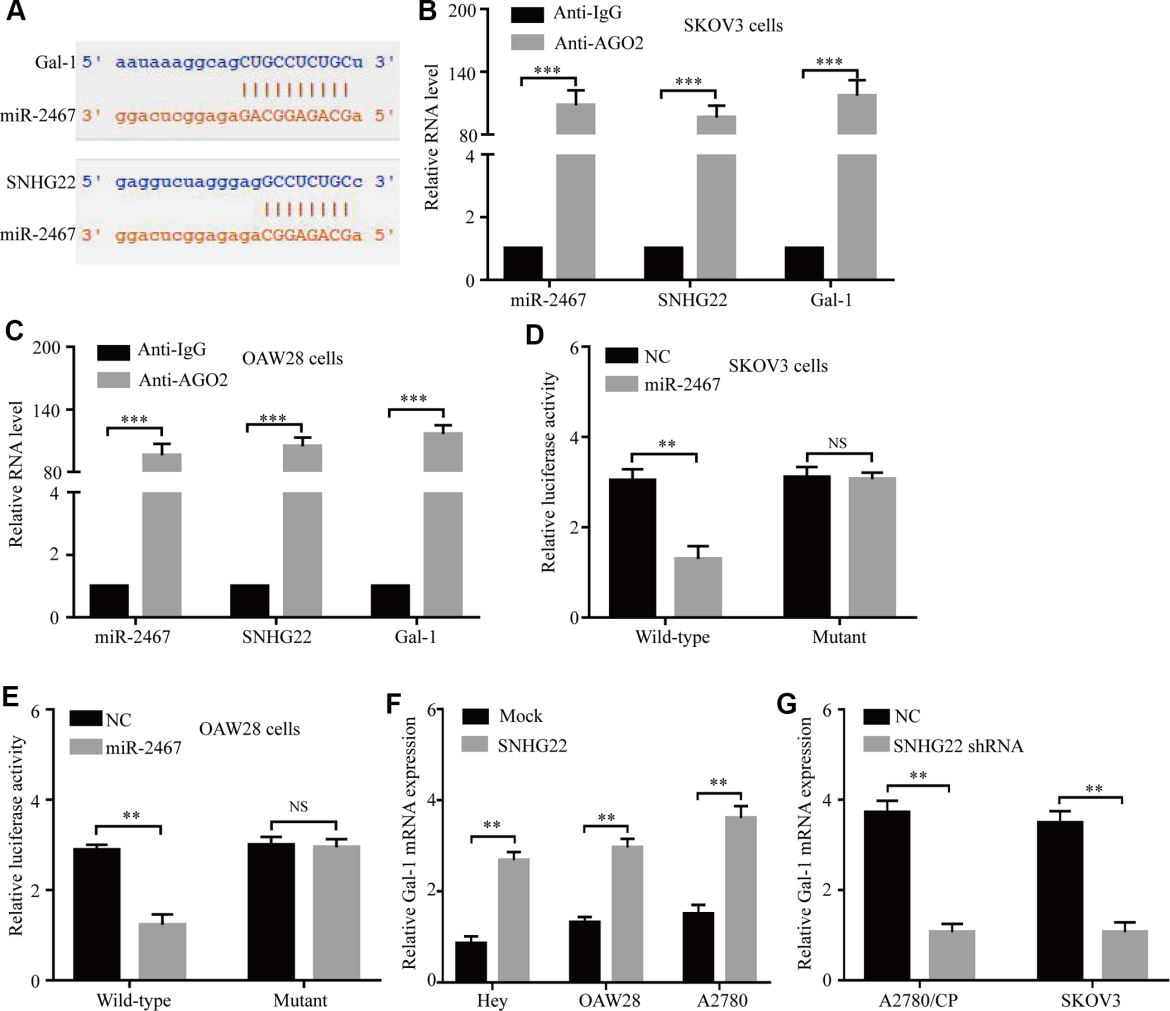
**SNHG22 functions as a ceRNA for miR-2467 to facilitate Gal-1 expression in EOC cells.** (**A**) Target sequences in SNHG22 and Gal-1 predicted to bind to miR-2467. (**B** and **C**) Anti-AGO2 RIP was performed in CAOV3 and OAW28 cells. The results indicated that SNHG22, miR-2467, Gal-1, and AGO2 formed a complex in SKOV3 and OAW28 cells. (**D** and **E**) Wild-type or mutated SNHG22 and Ga1-1 3′ UTRs were transfected into SKOV3 and OAW28 cells with synthetic miR-2467 or negative control (NC). Luciferase activity was detected 48 hours after transfection. (**F** and **G**) Gal-1 expression after forced or reduced SNHG22 expression was detected in CAOV3 cells by using qRT-PCR. Data are presented as the means ± SDs in three independent experiments. **P < 0.01, ***P < 0.001.

Therefore, miR-2467 attracted our attention, as it might directly target SNHG22 and Gal-1 (Figure 4A). Next, we explored whether SNHG22 could bind to miR-2467. We carried out an RNA immunoprecipitation (RIP) assay with an antibody against argonaute 2 (AGO2) in SKOV3 and OAW28 cells. The results showed that miR-2467, SNHG22, and Gal-1 were significantly enriched by the AGO2 antibody (Figure 4B and 4C). These results suggest that SNHG22 and Gal-1 may act as binding platforms for AGO2 and miRNAs. To further confirm these predictions, wild-type (wt) and mutant (mu) SNHG22 and Gal-1 3′ UTR luciferase reporter vectors (pLG3 vector) were used. Luciferase reporter vectors containing a wt or mu SNHG22/Gal-1 3′ UTR were cotransfected with miR-2467 mimics or a negative control into SKOV3 and OAW28 cells. The relative luciferase activity was significantly inhibited in cells cotransfected with the wt SNHG22/Gal-1 3′ UTR luciferase reporter and miR-2467 mimic compared with the negative control (NC) cells (Figure 4D and 4E). Moreover, Gal-1 mRNA expression was significantly increased after overexpression of SNHG22 (Figure 4F). In contrast, Gal-1 mRNA expression in the SNHG22 shRNA group was significantly reduced compared with that in the control cells (Figure 4G).

### Gal-1 expression in tumor tissues and its correlation with SNHG22 and miR-2467

**Figure 5:**
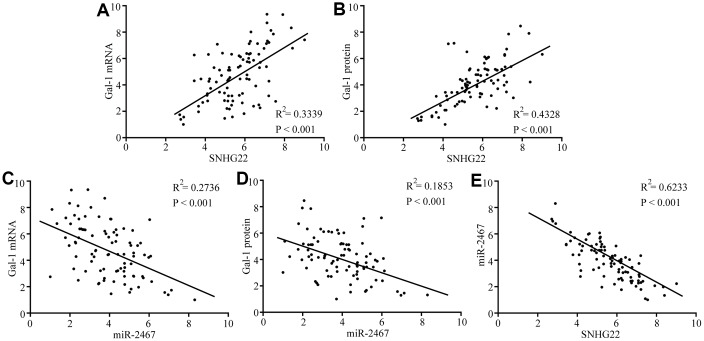
**Correlations between SNHG22, miR-2467, and Gal-1 were observed in EOC tissues**. (**A**) A positive correlation between SNHG22 and Gal-1 mRNA was observed in tumor tissues. (**B**) A positive correlation between SNHG22 and Gal-1 protein was observed in tumor tissues. (**C**) A negative correlation between miR-2467 and Gal-1 mRNA was observed in tumor tissues. (**D**) A negative correlation between miR-2467 and Gal-1 protein was observed in tumor tissues. (**E**) A negative correlation between miR-2467 and SNHG22 was observed in tumor tissues.

Next, we analyzed the expression of Gal-1 mRNA and protein expression in 90 EOC patient tissues. Spearman’s rank correlation analysis revealed that Gal-1 mRNA and protein expression was positively correlated with SNHG22 but negatively correlated with miR-2467 (Figure 5A–5E). These results further indicate that SNHG22 regulates miR-2467/Gal-1 -related signaling in EOC cells.

### Knockdown of Gal-1 reverses SNHG22-induced EOC chemotherapy resistance

**Figure 6:**
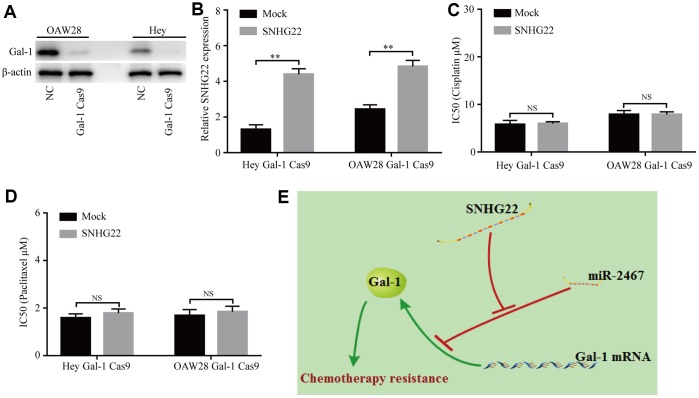
**Knockdown of Gal-1 reverses SNHG22-induced EOC chemotherapy resistance.** (**A**) Gal-1 expression in EOC cells was modified by CRISPR/Cas9. (**B**) SNHG22 expression in Gal-1-knockdown EOC cells was modified by cDNA transfection. (**C** and **D**) Cisplatin or paclitaxel sensitivity was measured using CCK-8 assays in Gal-1 knockdown EOC cells. (**E**) Working model: The forced expression of SNHG22 facilitates the chemotherapy resistance of EOC cells. Data are presented as the means ± SDs in three independent experiments. **P < 0.01.

We further determined whether SNHG22 regulates chemotherapy resistance of EOC cells in a Gal-1-dependent manner. CRISPR/Cas9-mediated Gal-1 knockdown was used in this study. Our results showed that the Gal-1-specific vector (pSpCas9) effectively knocked down Gal-1 expression in EOC cells (Figure 6A). Next, the transfection efficiencies of a lentivirus expressing SNHG22 and the mock control were confirmed using qRT-PCR in Gal-1 knockdown EOC cells (Figure 6B). The CCK-8 assay revealed that forced SNHG2 expression does not affect the chemotherapy sensitivity of Gal-1 knockdown EOC cells (Figure 6C and 6D). The results indicate that SNHG22 promotes chemotherapy resistance in EOC cells in a Gal-1-dependent manner (Figure 6E).

## DISCUSSION

Recently, an increasing number of reports have shown that dysregulated lncRNAs act as oncogenes or tumor suppress genes participating in cancer chemotherapy resistance [22, 24, 25]. Here, we demonstrated that the expression of SNHG22 was significantly increased in EOC tumor tissues. Knockdown of SNHG22 enhanced the sensitivity of EOC cells to cisplatin and paclitaxel. Our results reveal a novel mechanism regulating EOC chemoresistance. Cisplatin and paclitaxel are antitumor agents that are widely used as a first-line clinical therapeutic regimen for EOC [26]. However, their application is limited by primary or acquired drug resistance in EOC patients. To improve the efficiency of EOC prognosis, overcoming chemoresistance is important.

LncRNAs could act as ceRNAs to modulate the expression of target miRNAs in a variety of cancer cells and regulate tumor development and progression, including chemotherapy resistance [22, 24]. For example, forced expression of SCARNA2 significantly promoted chemoresistance via sponging miR-342-3p in CRC cells [22]. Increased lncRNA H19 expression induces bortezomib resistance in multiple myeloma by upregulating MCL-1 via sponging miR-29b-3p [24] LncR-D63785 acts as a competitive endogenous RNA of miR-422a and promotes chemotherapy resistance by blocking miR-422-dependent suppression of MEF2D in gastric cancer [27].

Several SNHG family members have been reported to act as oncogenes participating in the processes of tumor progression [7, 9, 28]. In this study, our data highlight a novel oncogenic function of SNHG22 in EOC chemotherapy resistance. Here, SNHG22 and Gal-1 have been predicted and confirmed as targets of miR-2467 using bioinformatics analysis, RIP assays and luciferase reporter assays. Gal-1 is considered one of the typical galectins and is overexpressed in many tumors, including EOC [5, 20]. Dysregulation of Gal-1 is involved in many biological processes, including immune evasion, immunoregulation, cell differentiation and proliferation, tissue development, premRNA splicing, and tumor progression [20, 29–31]. In our previous study, we found that Gal-1 was overexpressed in EOC tissues and that its tumor levels were positively correlated with poor clinical characteristics. Forced Gal-1 expression induces EOC cell resistance to cisplatin and activation of the H-Ras/Raf/ERK pathway [5]. Therefore, forced SNHG22 expression may be associated with the regulation of Gal-1, thus regulating the chemoresistance of EOC. The expression of Gal-1 in EOC cells with forced SNHG22 expression was dramatically increased, and the knockdown of SNHG22 significantly reduced the level of Gal-1, revealing the regulatory role of SNHG22 on Gal-1.

In summary, our results revealed that SNHG22, was upregulated in EOC and associated with chemotherapy resistance and poor prognosis of EOC patients. Mechanistically, we found that SNHG22 acts as a ceRNA to regulate Gal-1 expression by competitively binding to miR-2467. Taken together, our data suggest that SNHG22 might be a promising prognostic biomarker and therapeutic target for EOC.

## MATERIALS AND METHODS

### Cell lines and clinical tissues

Eight EOC cell lines (Hey, OAW28, COV-362, OVCAR-3, CAOV3, SKOV3, A2780, and A2780/CP) were used in this study, and tissue collection were performed as in our previous study [32]. In brief, EOC cells were purchased from the Institute of Biochemistry and Cell Biology, Chinese Academy of Sciences (Shanghai, China). All of the cells were cultured in Dulbecco’s modified Eagle’s medium (DMEM; Invitrogen, Carlsbad, CA) and supplemented with 10% fetal bovine serum (FBS) (Invitrogen) and penicillin/streptomycin (Invitrogen).

Human normal ovarian and EOC tissues were obtained along with written informed consent and pathology reports from the Obstetrics and Gynecology Hospital of Fudan University (from Jan. 2010 to Dec. 2013). Sample collection was performed after approval by the institutional ethics review committee of the Obstetrics and Gynecology Hospital of Fudan University. No patient had undergone chemotherapy before surgery. Surgical evaluation was used to determine the clinical stage of the cancer and the presence of metastases, whereas histopathologic analysis was performed by gynecologic pathologists to assess cancer type and grade.

### Quantitative real-time polymerase chain reaction, immunohistochemistry, and western blotting

Quantitative real-time polymerase chain reaction (qRT-PCR), immunohistochemistry (IHC) and western blotting were performed as described in our previous studies and as described in the Supplementary Materials and Methods [5, 20, 22]. The primers and antibodies used in this study are listed in Supplementary Tables 1 and 2.

### Transfection experiment

The transfection experiments are described in the Supplementary Materials and Methods. The target sequences of shRNAs are listed in Supplementary Table 3.

### Drug sensitivity assay

Cells were seeded in 96-well plates at densities of 2 × 10^3^ cells per well. Twenty-four hours later, the cells were placed in complete medium containing various concentrations of cisplatin (1, 5, 25, 100, or 500 μM) or paclitaxel (0.2, 1, 5, 25, or 100 μM). After 72 hours, the sensitivity of the cells to cisplatin or paclitaxel was measured using a Cell Counting (CCK-8) Kit (Dojindo, Cat No.CK04).

### RNA immunoprecipitation (RIP) assay

The RIP assay was performed with the EZ-Magna RIP Kit (Millipore, Bedford, MA, USA) and an AGO2 antibody (Abcam, Cambridge, United Kingdom) as proviously described [33]. qRT-PCR was performed to detect coprecipitated RNA expression.

### CRISPR/Cas9-mediated Gal-1 knockdown

The CRISPR/Cas9 double vector lentiviruses for Ga1-1 knockdown were purchased from GeneChem Corporation (Shanghai, China). Hey and OAW28 cells were transduced with lentiviruses as previously described [34]. Gal-1 expression was detected by western blotting.

### Luciferase reporter assay

Generation of the mutant SNHG22 and Gal-1 3′ UTR luciferase reporter vectors was carried out by using a Mutagenesis Kit (QIAGEN, California, USA) according to the manufacturer’s instructions. Hey cells were seeded into 96-well plates and cotransfected with a luciferase reporter vector and miR-2467 mimics or a negative control using Lipofectamine 2000 according to the manufacturer’s instructions (Invitrogen, California, USA). After 48 hours, the firefly and Renilla luciferase activities were quantified with a dual-luciferase reporter assay (Promega, Wisconsin, USA).

### Statistical analysis

Statistical analysis was performed with SPSS software (19.0; SPSS, Inc., Chicago, IL) as previously described [20, 22]. All data are presented as the mean ± SD. P < 0.05 was considered statistically significant.

## Supplementary Material

SUPPLEMENTARY MATERIALS

Supplementary Tables
