# Supplementary material for: SNHG22 overexpression indicates poor prognosis and induces chemotherapy resistance via the miR-2467/Gal-1 signaling pathway in epithelial ovarian carcinoma
Source: Aging (Albany NY). 2019 Oct 3;11(19):8204–16. doi: 10.18632/aging.102313 (PMC6814594; doi:10.18632/aging.102313)
Supplement: SUPPLEMENTARY MATERIALS [file aging-11-102313-s001.pdf]

SUPPLEMENTARY MATERIALS

qRT-PCR, immunohistochemistry (IHC), and western blotting

For qRT-PCR, total RNA was extracted with the TRIzol Kit (Invitrogen) according to the manufacturer's instructions. qRT-PCR was performed using a SYBR Green PCR kit (TaKaRa, Otsu, Japan), according to the manufacturer's instructions. The All-in-One^{TM} miRNA qRT-PCR detection kit (GeneCopoeia, Rockville, MD, USA) was used to determine miR-2467 expression.

For IHC, paraffin-embedded tissue sections (4 μm) were prepared using classical methods and Gal-1 expression was detected using an immunoperoxidase method. According to the intensity and total area of the staining, Gal-1 expression levels were classified as either high (>20% of tumor section) or low (<20% of tumor section) using an integrated imaging system (MetaMorph Imaging System version 3.0; Universal Imaging Corp, Buckinghamshire, UK). A positive reaction for Gal-1 expression was scored in 1 to 10 grade categories depending on the intensity of the staining (0 to 100%). The correlation analysis was performed among Gal-1 and SNHG22/miR-2467.

For western blotting, Cell lysates were collected and centrifuged for 15 min at 12 000 r.p.m., 4 °C. The supernatant was transferred to a clean tube and proteins concentrations were then quantified using the BCA Kit (Pierce, Rockford, IL, USA). Proteins were then separated on SDS-PAGE gels and transferred to nitrocellulose membranes. The membranes were then blocked with 5% skim milk for 2 h at room temperature and incubated overnight at 4 °C with primary antibodies. Immune complexes were then detected by incubating the nitrocellulose membranes with HRP-conjugated goat anti-mouse/rabbit antibody (Beyotime, Shanghai, China) for 2 h at room temperature, followed by exposure of the membrane to enhanced chemiluminescence reagents (Pierce, Rockford).

Transfection experiment

To overexpression or knockdown the expression of SNHG22, SNHG22 cDNA and short hairpin RNA interference vector lentiviruses were constructed by GeneChem Corporation (Shanghai, China). The nucleotide sequences of shRNAs for SNHG22 are listed in Supplementary Table 3.

The miR-2467 mimic was purchased from GenePharma (Shanghai, China). EOC cells were transfected with Lipofectamine 2000 (Invitrogen) according to the manufacturer's instructions.
