# Supplementary material for: SNHG22 overexpression indicates poor prognosis and induces chemotherapy resistance via the miR-2467/Gal-1 signaling pathway in epithelial ovarian carcinoma
Source: Aging (Albany NY). 2019 Oct 3;11(19):8204–16. doi: 10.18632/aging.102313 (PMC6814594; doi:10.18632/aging.102313)
Supplement: Supplementary Tables [file aging-11-102313-s002.pdf]

SUPPLEMENTARY TABLES

Supplementary Table 1. The primers used in this study.

Gene	Forward primer (5′-3′)	Reverse primer(5′-3′)
SNHG22	AGGAGAGCTGCTCTTCACAGG	TCCTAGGCTGAGTGTGTCTCC
Gal-1	CTCCTGACGCTAAGAGCTTCG	CCAGGCTGGAAGGGAAAGAC
GAPDH	CCTGGCACCCAGCACAAT	GGGCCGGACTCGTCATCG

Supplementary Table 2. The antibodies used in this study.

Antibody	Company	Cat No.
Gal-1	Abcam	ab138513
GAPDH	Abcam	ab181602
HRP-labeled Goat Anti-Rabbit IgG(H+L)	Beyotime	A0208
HRP-labeled Goat Anti-mouse IgG(H+L)	Beyotime	A0216
AGO2	Abcam	ab32381
IgG	Abcam	ab172730

Supplementary Table 3. The target sequences of shRNAs for SNHG22.

shRNA	Target sequence
SNHG22 shRNA -1	GTGGGCAAGTGAGATTATC
SNHG22 shRNA -2	GGTCTCCTGTGAACCCTGA
SNHG22 shRNA -3	GATCAGCCTGTTGACAGGA
